# Spectrum of psychiatric adverse reactions to cyclin‐dependent kinases 4/6 inhibitors: A pharmacovigilance analysis of the FDA adverse event reporting system

**DOI:** 10.1111/cns.14862

**Published:** 2024-07-15

**Authors:** Zhijun Xiao, Jinming Cao, Shenghong Wu, Ting Zhou, Canye Li, Jingjing Duan, Zhen Yang, Feng Xu

**Affiliations:** ^1^ Department of Pharmacy Shanghai University of Medicine & Health Sciences Affiliated Sixth People's Hospital South Campus Shanghai China; ^2^ Department of Oncology Shanghai University of Medicine & Health Sciences Affiliated Sixth People's Hospital South Campus Shanghai China; ^3^ Department of Central Laboratory Shanghai University of Medicine & Health Sciences Affiliated Sixth People's Hospital South Campus Shanghai China

**Keywords:** cyclin‐dependent kinases 4/6 inhibitors, drug safety, FAERS, pharmacovigilance, psychiatric disorder

## Abstract

**Background:**

The emergence of cyclin‐dependent kinases 4/6 inhibitors (CDK4/6i) represented a major breakthrough in the treatment of breast cancer over the past decade. In both clinical trials and real‐world settings, it was observed that patients using CDK4/6i might experience psychiatric adverse events (PAEs). Herein, we conducted a pharmacovigilance study to comprehensively assess the correlation between CDK4/6i and PAEs.

**Method:**

We obtained individual case safety reports submitted to the FDA Adverse Events Reporting System (FAERS) during the period from January 2015 to December 2023. In disproportionality analysis, the reporting odds ratio (ROR) and information component (IC) values were calculated for each adverse event‐drug combination. Univariate logistic regression analysis was utilized to explore factors associated with PAEs following CDK4/6i treatment.

**Results:**

A total of 95,591 reports related to CDK4/6i were identified, with 6.72% reporting PAEs, and this proportion exhibited an annual upward trend. Based on the ROR and IC values, 17 categories of PAEs were defined as CDK4/6i‐related PAEs. Among these PAEs, insomnia, stress, eating disorder, depressed mood, and sleep disorder were very common, each accounting for over 10% of CDK4/6i reports. Ribociclib showed the highest risk signal of CDK4/6i‐related PAEs (ROR = 1.89[1.75–2.04], IC_025_ = 0.79), followed by palbociclib (ROR = 1.47[1.41–1.53], IC_025_ = 0.49), while abemaciclib did not exhibit a significant signal (ROR = 0.52[0.44–0.62], IC_025_ = −1.13). Female sex, younger age and weight exceeding 80 kg were significant risk factors for the incidence of CDK4/6i‐related PAEs.

**Conclusions:**

Using data from a real‐world, large‐scale spontaneous reporting system for adverse drug reactions, our study delineated the spectrum of PAEs to CDK4/6i. This potentially offered valuable insights for healthcare professionals to manage the risk of PAEs in patients receiving CDK4/6i treatment, particularly those with psychiatric disorders.

## Graphical abstract

This is a pharmacovigilance study to comprehensively assess the correlation between cyclin‐dependent kinases 4/6 inhibitors (CDK4/6i) and psychiatric adverse events (PAEs). Our study identified 17 categories of PAEs highly associated with CDK4/6i through disproportionality analysis, and revealed differences in the profiles of PAEs among three CDK4/6i.

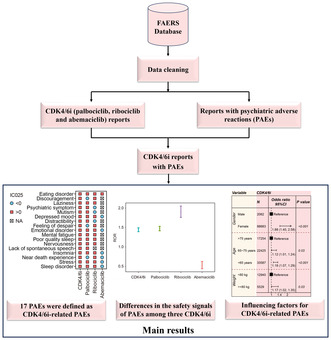

## 1 INTRODUCTION

Breast cancer is the most frequently diagnosed cancer worldwide and ranks as the fifth leading cause of cancer‐related deaths, with 684,996 fatalities in 2020.^1^ Hormone receptor‐positive (HR^+^)/human epidermal growth factor receptor 2‐negative (HER2^−^) breast cancer is the most common subtype of breast cancer, accounting for 68% of total breast cancer cases.^2^ Endocrine therapy (ET) serves as the primary systemic treatment for HR^+^ breast cancer, inhibiting tumor growth by specifically addressing their reliance on estrogen signaling.^3^ In the last decade, the development of cyclin‐dependent kinases 4/6 inhibitors (CDK4/6i) was a major breakthrough for HR^+^/HER2^−^ breast cancer treatment.^4^

Palbociclib, ribociclib, and abemaciclib, three CDK4/6i, have been respectively validated for their efficacy and safety in treating breast cancer through landmark phase 3 clinical trials, namely PALOMA‐2,^5^ MONALEESA‐2,^6^ and MONARCH‐2.^7^ In these trials, the combination of CDK4/6i with ET significantly prolonged progression‐free survival for patients with HR^+^/HER2^−^ metastatic breast cancer compared to ET alone. Thus, CKD4/6i have been approved as first‐line treatment in combination with ET for patients with HR^+^/HER2^−^ metastatic breast cancer. The primary anti‐cancer mechanism of CDK4/6i is suppression of RB phosphorylation, enforcing G1 cell cycle arrest, thus inhibiting cancer cell proliferation.^8^ The most common toxicities of CDK4/6i include neutropenia, diarrhea, nausea and fatigue.^9^

There was substantial evidence suggesting that breast cancer patients have an increased risk of psychiatric disorders, including anxiety, depression, stress‐related disorders, suicide, as well as sleep disturbance and sexual dysfunctions.^10^ These conditions negatively impact patients' survival, recurrence, and quality of life.^11^, ^12^, ^13^ Preclinical study also suggested that psychological stress induces tumor immune evasion and promotes the progression of breast cancer.^14^ Consequently, it is imperative to comprehend the psychiatric safety profile of breast cancer treatment for optimizing patient management. Psychiatric adverse reactions (PAEs) are a category of adverse events (AEs) associated with CDK4/6i treatment. In the three landmark studies of CDK4/6i, insomnia was the most common PAE, with an incidence rate ranging from 9.52% to 16.22%.^5^, ^6^, ^7^ Other PAEs such as depression, anxiety, confusional state, psychotic disorder, suicidal ideation and metal status changes have also been noted in patients receiving CDK4/6i in these trials. In the findings from a multi‐country survey, PAEs including low sexual interest, anxiety, and insomnia occurred in 50%, 34%, and 14% of patients on CDK4/6i treatment, respectively, severely affecting their quality of life.^15^ Based on data from VigiBase, the WHO global database of individual case safety reports, a previous study indicated that ribociclib and palbociclib were associated with depression.^16^ A case report documented that ribociclib caused visual hallucinations in a patient with metastatic breast cancer.^17^ However, it remains unclear whether additional PAEs are associated with CDK4/6i. Furthermore, while the efficacy of the three CDK4/6i is similar, there are some differences in the toxicity profiles among these agents. Study has documented that abemaciclib exhibiting a higher incidence of gastrointestinal toxicities, while palbociclib and ribociclib show higher rates of hematological toxicities.^18^ Nevertheless, there is currently no research elucidating the differences in psychiatric safety profiles among the three CDK4/6i. Due to the low incidence of certain PAEs, it is challenging to obtain evidence about their association with CDK4/6i in clinical trials. To address this issue, conducting a post‐marketing pharmacovigilance study using data from real‐world, large‐scale spontaneous reporting system may offer a feasible approach to uncover such evidence.

The FDA Adverse Events Reporting System (FAERS) is the primary post‐marketing surveillance system in the US, responsible for collecting and storing spontaneous AE reports submitted by drug manufacturers, healthcare professionals, and consumers. This database contains over 28 million reports submitted to the FDA from 1969 to 2023. Safety signals identified from the FAERS have prompted numerous drug safety communications and boxed warnings, potentially altering patient drug treatments, physician prescribing habits, and insurance coverage decisions.^19^ In this study, based on the FAERS, we conducted a pharmacovigilance analysis to characterize and evaluate PAEs associated with CDK4/6i, aiming to provide valuable references for healthcare professionals, patients and researchers.

## 2 METHODS

### 2.1 Data source

Individual case safety reports submitted between January 2015 and December 2023 were obtained from the FAERS (https://fis.fda.gov/extensions/FPD‐QDE‐FAERS/FPD‐QDE‐FAERS.html) and were subjected to data cleaning. The FAERS consolidates safety reports for each quarter into a packaged file. Within each packaged file, the detailed information regarding all case reports for that quarter is organized across seven distinct tables. These tables include data covering patient demographics and administrative information (DEMO), medication and biological products administered (DRUG), adverse drug reactions (REAC), patient outcomes (OUTC), report sources (RPSR), drug therapy start and end dates (THER), and indications for medication use (INDI). The AE terms in the FAERS are standardized to the preferred term (PT) within the Medical Dictionary for Regulatory Activities (MedDRA). The hierarchical structure of MedDRA comprises five levels, i.e., lowest level term (LTT), PT, high level term (HLT), high level group term (HLGT), and system organ class (SOC). These levels were arranged in a sequence from the most specific to the most general. Each PT was classified into a primary SOC, and it could also be classified into one or more secondary SOCs. In this study, a total of 571 PTs corresponding to PAEs were extracted from MedDRA (version 26.1), with the primary SOC of these PTs being psychiatric disorders.

### 2.2 Identification of CDK4/6i reports with psychiatric AEs

We implemented a comprehensive four‐step analysis to identify potential PAEs associated with CDK4/6i. Firstly, we eliminated duplicate reports obtained from the FAERS database. For reports with the same CASEID, we retained only the latest report, which contain the most complete information. Besides, we conducted an extra deduplication step, excluding cases with identical gender, age, reporting country, event date, AEs, and prescribed drugs.^20^, ^21^ Secondly, we screened CDK4/6i reports by matching the generic and brand names of CDK4/6i in the DRUG file. The FAERS categorizes the role of each drug in its associated reports as Primary Suspect (PS), Secondary Suspect (SS), Concomitant (C), or Interacting (I). We exclusively selected reports where CDK4/6i were categorized as the PS, as the AEs in these reports were considered most likely to be associated with CDK4/6i when compared to other drugs. Thirdly, we extracted all reports containing PAEs from the REAC files. Finally, we identified CDK4/6i reports with PAEs by taking the intersection of PRIMARYID between the CDK4/6i reports and the reports containing PAEs.

### 2.3 Pharmacovigilance signal detection

Disproportionality analysis is widely utilized in pharmacovigilance studies to identify safety signals and generate hypotheses about potential causal relationships between drugs and AEs.^22^ In this study, the reporting odds ratio (ROR) and information component (IC), two frequently used quantitative measures in disproportionality analysis,^23^ were employed to quantify signals of PAEs in CDK4/6i reports. The ROR value and its 95% confidence interval (CI) were calculated as follows:

$$\mathrm{ROR}=\left(a/b\right)/\left(c/d\right);95\%\mathrm{CI}=\exp\left(\ln\left(\mathrm{ROR}\right)\pm 1.96\sqrt{1/a+1/b+1/c+1/d}\right)$$

where *a* is the number of cases with suspect AEs of interest drug, *b* is the number of cases with suspect AEs of all other drug, *c* is the number of cases with other AEs of interest drug, and *d* is the number of cases with other AEs of all other drug.

The IC value and its lower limit of the 95%CI (IC_025_) were calculated by using R package *pvm*. A pharmacovigilance signal was considered significant, indicating a strong association between the suspected AE and the interest drug when the number of suspected AE was three or more, with the lower limit of the 95% CI for the ROR was greater than one^21^ and the IC_025_ was greater than zero.^24^ If a significant signal was observed between a PAE and CDK4/6i, that PAE was defined as CDK4/6i‐related PAE.

### 2.4 Time‐to‐onset analysis

The cases with completed START_DT (a date therapy was started) in the THER files and EVENT_DT (a date the AEs occurred) in the DEMO files were used for the time‐to‐onset analysis. Given the necessity to consider right truncation when estimating the time‐to‐onset of AEs from spontaneous reporting databases,^25^ we established an analysis period of 365 days following the initiation of drug administration. The Weibull Shape Parameter (WSP) test was used for statistical analysis of time‐to‐onset,^26^ describing the probability of AE increase or decrease over time. When the shape parameter *β* is <1 and its 95% CI <1, it implies a decreasing risk of AE occurrence over time; if *β* is close to 1 with a 95% CI including 1, it suggests a constant risk; and when *β* >1 with a 95% CI >1, it indicates an increasing risk over time.^27^

### 2.5 Statistical analysis

Statistical analysis in this study was conducted using R software (version 4.3.0). Descriptive analysis was employed to summarize the demographic and administrative characteristics of cases occurred CDK4/6i‐related PAEs. Univariate logistic regression analysis was used to calculate the odds ratio (OR) for the occurrence of CDK4/6i‐related PAEs under different demographic characteristics, including age, gender, and weight. Mann–Whitney *U* test was used to compare the median time to onset between different subgroups. A *p* value less than 0.05 was considered statistically significant.

## 3 RESULTS

### 3.1 PAEs among CDK4/6i users in the FAERS from 2015 to 2023

**FIGURE 1:**
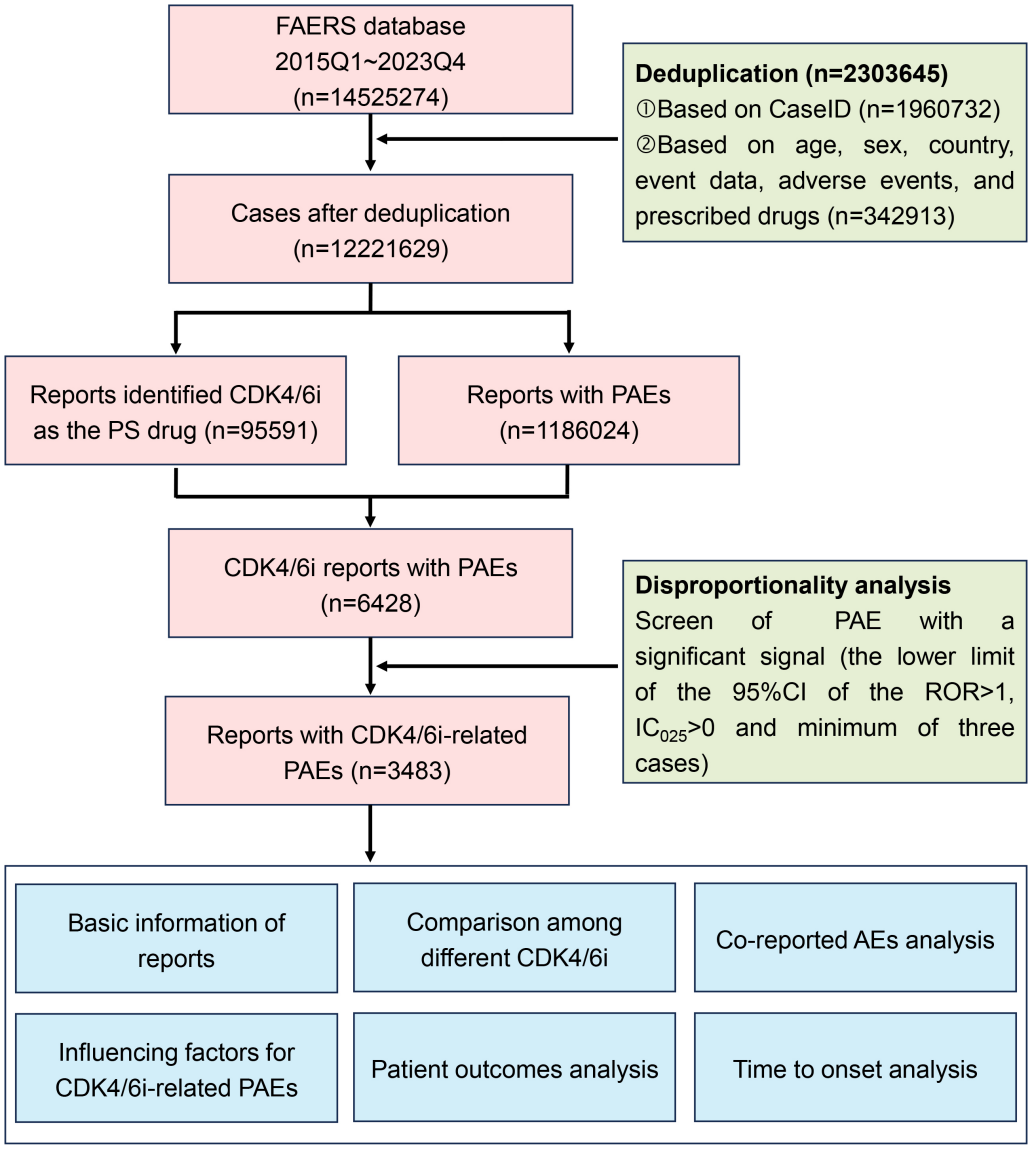
Flowchart illustrating the analysis process of this study.

The comprehensive data processing workflow of this study was illustrated in Figure 1. A total of 12,221,629 non‐duplicated reports submitted between January 2015 and December 2023 were obtained. Among them, 95,591 reports listed CDK4/6i as the PS drug, and there were 1,186,024 cases experiencing PAEs. Subsequently, an intersection analysis revealed that 6428 cases of CDK4/6i reported PAEs.

**FIGURE 2:**
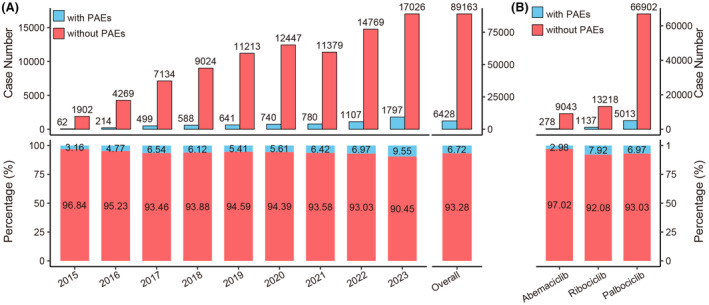
Statistics of CDK4/6i reports with and without PAEs in the FAERS from 2015 to 2023. (A) The upper bar chart shows the annual number of CDK4/6i reports with and without PAEs, along with the overall situation over 9 years. The proportional bar chart below indicates the annual proportions of CDK4/6i reports with and without PAEs, as well as the overall situation over 9 years. (B) The upper bar chart shows the number of reports with and without PAEs for different CDK4/6i. The proportional bar chart below indicates the proportions of reports with and without PAEs for different CDK4/6i.

We calculated the annual count and proportion of CDK4/6i reports with PAEs. As shown in Figure 2A, the number of CDK4/6i reports with PAEs exhibited a gradual increase from 2015 to 2023. The proportion of CDK4/6i reports with PAEs among all CDK4/6i reports also showed an increasing trend, rising from 3.16% (62/1964) in 2015 to 9.55% (1797/18,823) in 2023, with a total proportion of 6.72% (6428/95,591). The proportion of reports with PAEs differed among the three CDK4/6i. To be specific, the percentage of reports with PAEs for ribociclib was 7.92% (1137/14,355), whereas for abemaciclib, it was 2.98% (278/9321) (Figure 2B).

### 3.2 Identification of CDK4/6i‐related PAEs

**FIGURE 3:**
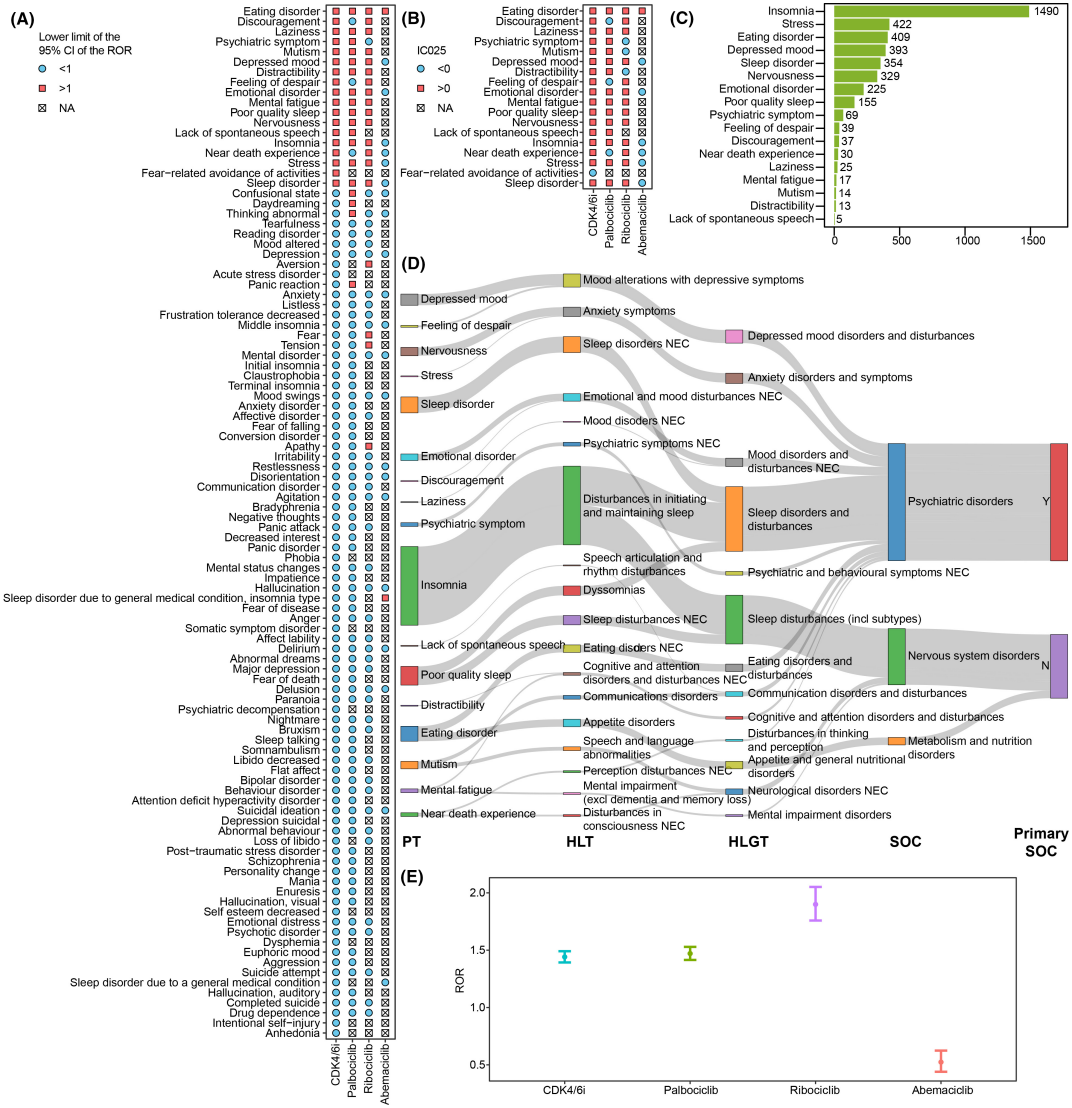
Identification of CDK4/6i‐related PAEs based on the FAERS database. (A) The heatmap shows the ROR values for 102 PAEs reported in CDK4/6i reports. The red square and the blue circle respectively indicate the lower limit of the 95% CI for ROR being greater than 1 and less than 1, and the gray square with a cross indicates that the ROR value could not be calculated due to fewer than three reports. (B) The heatmap shows the IC_025_ values for 18 PAEs. (C) The bar chart displays the number of reports for each of the CDK4/6i‐related PAEs. (D) The Sankey diagram illustrates the relationship between the PTs and other hierarchies in MedDRA. (E) Forest plot shows the ROR values of CDK4/6i‐related PAEs.

The case number for each PAE in CDK4/6i reports was listed in Tables S1 and S2. Insomnia, anxiety, depression, confusional state, and stress were the top five PAEs with the highest number of cases, reported in 1490 (23.18%), 1014 (15.77%), 828 (12.88%), 685 (10.66%), and 422 (6.57%) cases, respectively. The ROR values were calculated for PAEs with a minimum of three cases, utilizing the entire FAERS database as a comparator (Table S2). There were differences in the safety signals of PAEs among the three CDK4/6i (Figure 3A). Specifically, ribociclib and palbociclib had 19 and 18 PAEs with significant risk signals, respectively, while abemaciclib had only two. We calculated the IC_025_ values for 18 PAEs and found that the IC_025_ value of fear‐related avoidance of activities was less than zero (Figure 3B). Overall, according to the defined criteria, 17 PAEs were identified as CDK4/6i‐related PAEs. Among them, insomnia and lack of spontaneous speech had the highest and lowest numbers of cases respectively (Figure 3C), while eating disorder and sleep disorder had the highest and lowest ROR values respectively (Table S2).

The affiliation of the PTs for CDK4/6i‐related PAEs with other hierarchies in MedDRA was illustrated in Figure 3D. Insomnia, poor quality sleep, mutism, mental fatigue and near death experience were secondary to the SOC nervous system disorders, while eating disorder was secondary to the SOC metabolism and nutrition disorders. The ROR value for CDK4/6i‐related PAEs was calculated based on 3483 cases occurred CDK4/6i‐related PAEs. The CDK4/6i treatment was significantly associated with the occurrence of CDK4/6i‐related PAEs (ROR = 1.44[1.39–1.49], IC_025_ = 0.47) (Figure 3E). Besides, there were differences in safety signals among three CDK4/6i in respect to CDK4/6i‐related PAEs, with ribociblib displaying the highest risk signal (ROR = 1.89 [1.75–2.04], IC_025_ = 0.79), followed by palbociclib (ROR = 1.47[1.41–1.53], IC_025_ = 0.49), while abemaciclib did not show a significant signal (ROR = 0.52[0.44–0.62], IC_025_ = −1.13).

### 3.3 Characteristics of reports with CDK4/6i‐related PAEs

The demographic characteristics of 3483 reports with CDK4/6i‐related PAEs were listed in Table 1. Palbociclib had the highest proportion of reports (76.80%), followed by ribociclib (19.55%) and abemaciclib (3.65%). Age data was available for 3035 reports, with the median age of patients being 65 years (Q1–Q3: 57.00–73.00). Dividing the patients into three different age groups using 65 and 75 years as cutoffs, we observed that the number of cases younger than 65 years was higher than that of the other two groups. Weight data was available for only 26.04% of cases, with a median patient weight of 71.20 kg (Q1–Q3: 60.00–84.82). Due to the predominance of breast cancer in females, nearly all cases were women (97.88%). Interestingly, the cases reported by consumers accounted for 57.91%, surpassing those reported by health professionals. Among the reporting countries, the highest number of cases (76.20%) was reported from the USA, followed by Brazil (3.62%) and Argentina (3.56%).

**TABLE 1:** Characteristics of 3483 cases with CDK4/6i‐related PAEs.

Characteristics	CDK4/6i *N* = 3483	Palbociclib *N* = 2675	Ribociclib *N* = 681	Abemaciclib *N* = 127
Age [Median (Q1–Q3)] (years)	65.00 [57.00, 73.00]	66.00 [58.00, 74.00]	60.00 [49.00, 69.00]	63.00 [52.00, 71.00]
Age group [*n* (%)]				
<65 years	1462 (41.98)	1188 (44.41)	241 (35.39)	33 (25.98)
65 ~ 75 years	930 (26.70)	822 (30.73)	93 (13.66)	15 (11.81)
>75 years	643 (18.46)	581 (21.72)	54 (7.93)	8 (6.30)
Unknown	448 (12.86)	84 (3.14)	293 (43.02)	71 (55.91)
Weight [Median (Q1–Q3)] (kg)	71.20 [60.00, 84.82]	73.00 [61.23, 86.00]	65.50 [57.75, 78.00]	64.41 [56.25, 68.85]
Weight group [*n* (%)]				
<80 kg	606 (17.39)	465 (17.38)	131 (19.24)	10 (7.87)
>=80 kg	301 (8.64)	264 (9.87)	37 (5.43)	0 (0.00)
Unknown	2576 (73.96)	1946 (72.75)	513 (75.33)	117 (92.13)
Gender				
Female	3409 (97.88)	2614 (97.72)	673 (98.83)	122 (96.06)
Male	43 (1.23)	40 (1.50)	2 (0.29)	1 (0.79)
Unknown	31 (0.89)	21 (0.79)	6 (0.88)	4 (3.15)
Reporter [*n* (%)]				
Consumer	2017 (57.91)	1386 (51.81)	537 (78.85)	94 (74.02)
Health‐professional	1442 (41.40)	1276 (47.70)	136 (19.97)	30 (23.62)
Unknown	24 (0.69)	13 (0.49)	8 (1.17)	3 (2.36)
Reporting country [*n* (%)]				
USA	2654 (76.20)	2429 (90.80)	114 (16.74)	111 (87.40)
Brazil	126 (3.62)	1 (0.04)	123 (18.06)	2 (1.57)
Argentina	124 (3.56)	90 (3.36)	34 (4.99)	0 (0.00)
Colombia	87 (2.50)	29 (1.08)	58 (8.52)	0 (0.00)
India	70 (2.01)	66 (2.47)	4 (0.59)	0 (0.00)
Others	318 (9.13)	59 (2.21)	245 (35.98)	14 (11.02)
Unknown	104 (2.99)	1 (0.04)	103 (15.12)	0 (0.00)

### 3.4 Co‐reported AEs and influencing factors for CDK4/6i‐related PAEs

**FIGURE 4:**
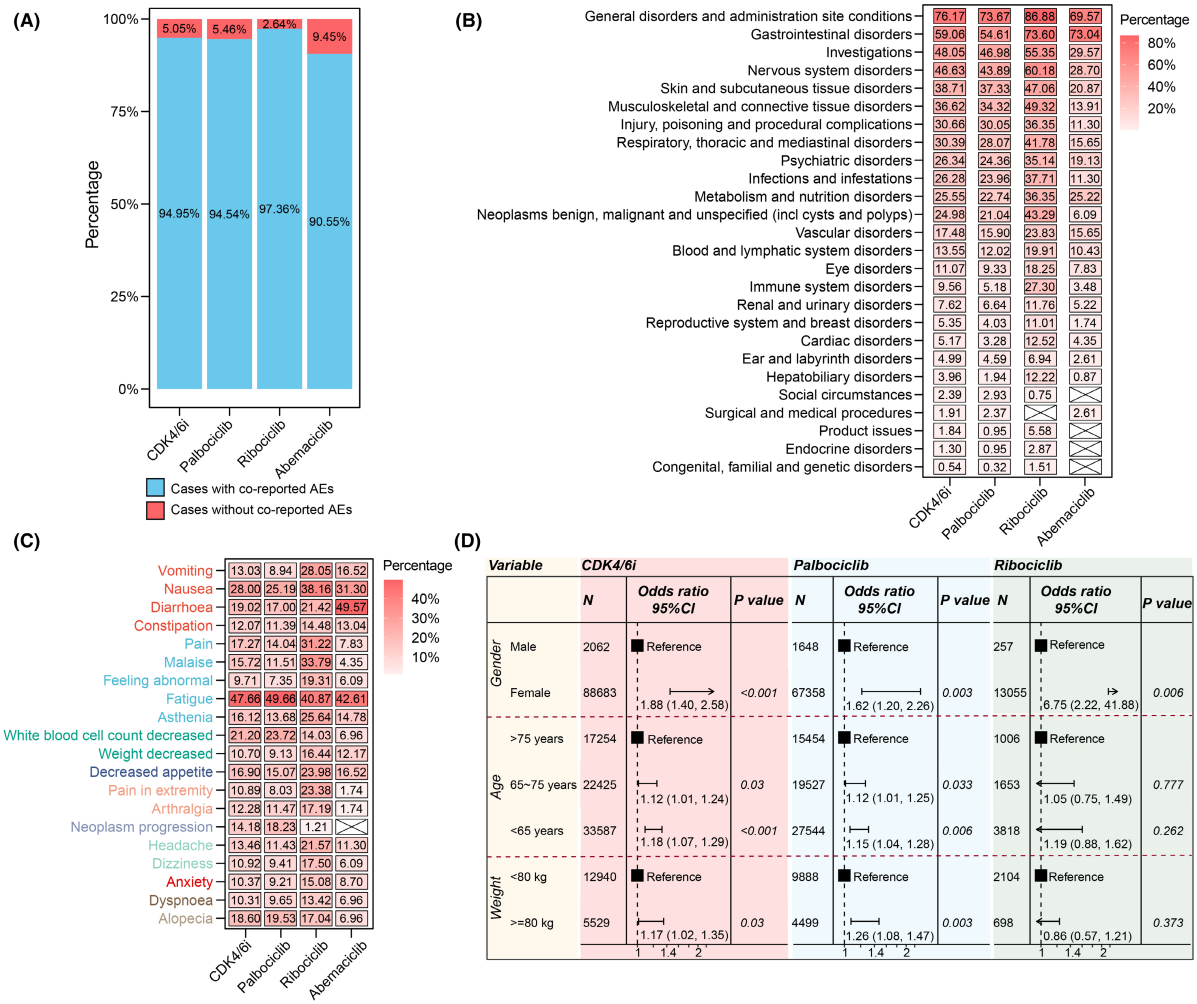
Co‐reported AEs and influencing factors for CDK4/6i‐related PAEs. (A) Bar plot shows the proportion of reports with and without co‐reported AEs in reports with CDK4/6i‐related PAEs. (B) Heatmap shows the statistics of SOC corresponding to the PTs of co‐reported AEs. (C) Heatmap illustrates statistics for the top 20 co‐reported AEs. The PTs with the same color belongs to the same SOC. (D) Forest plot shows the outcomes of univariate logistic regression analysis concerning demographic factors influencing CDK4/6i‐related PAEs.

We reviewed the co‐reported AEs associated CDK4/6i‐related PAEs. Out of the 3483 reports we examined, 94.95% were accompanied by the report of additional AEs, while 5.05% solely reported CDK4/6i‐related PAEs (Figure 4A). We classified the co‐reported AEs by SOC and found that among all 3307 cases with co‐reported AE, general disorders and administration site conditions, gastrointestinal disorders, and investigations were the three most frequently co‐reported AEs, reporting in 76.17%, 59.06%, and 48.05% of cases respectively (Figure 4B). Additionally, fatigue, nausea and white blood cell count decreased were the top three co‐reported AEs, occurring in 47.66%, 28.00%, and 21.20% of cases, respectively (Figure 4C). The co‐reports AEs for the three CDK4/6i exhibited certain differences. In palbociclib and ribociclib reports, fatigue had the highest co‐reported proportion, while in abemaciclib reports, diarrhea had the highest co‐reported proportion. Additionally, the co‐reported proportion of white blood cell count decreased for abemaciclib was lower than the other two CDK4/6i.

We further investigated demographic factors that might influence the occurrence of CDK4/6i‐related PAEs by univariate logistic regression analysis based on the total CDK4/6i reports. The distributions of age, gender, and weight among cases with and without CDK4/6i‐related PAEs were shown in Figures S1 and S2. In the reports of ribociclib and abemaciclib, over 50% of the reports lacked age information, while for all three drugs, over 70% of the reports lacked weight information. In all CDK4/6i cases, female patients had a higher risk of experiencing CDK4/6i‐related PAEs compared to males (OR = 1.88 [1.40, 2.58], *p* < 0.001) (Figure 4D). Compared to patients aged over 75 years, those aged 65 ~ 75 had 1.12 times higher odds of CDK4/6i‐related PAEs (OR = 1.12 [1.01–1.24], *p* = 0.03), and those under 65 has 1.18 times higher odds (OR = 1.18 [1.07–1.29], *p* < 0.001). Patients weighing over 80 kg were 1.17 times more likely to experience CDK4/6i‐related PAEs compared to those weighing less than 80 kg (OR = 1.17 [1.02–1.35], *p* = 0.03). We conducted separate analyses for the three CDK4/6i and found that age and weight were not influencing factors for CDK4/6i‐related PAEs occurrence in ribociclib. Since no cases of abemaciclib experiencing CDK4/6i‐related PAEs had a weight less than 80 kg among those with available weight information, we only analyzed gender and age for abemaciclib cases. Our analysis revealed that neither gender nor age were influencing factors for the occurrence of CDK4/6i‐related PAEs in abemaciclib (Figure S2).

### 3.5 Outcomes of cases with CDK4/6i‐related PAEs and time‐to‐onset analysis

**FIGURE 5:**
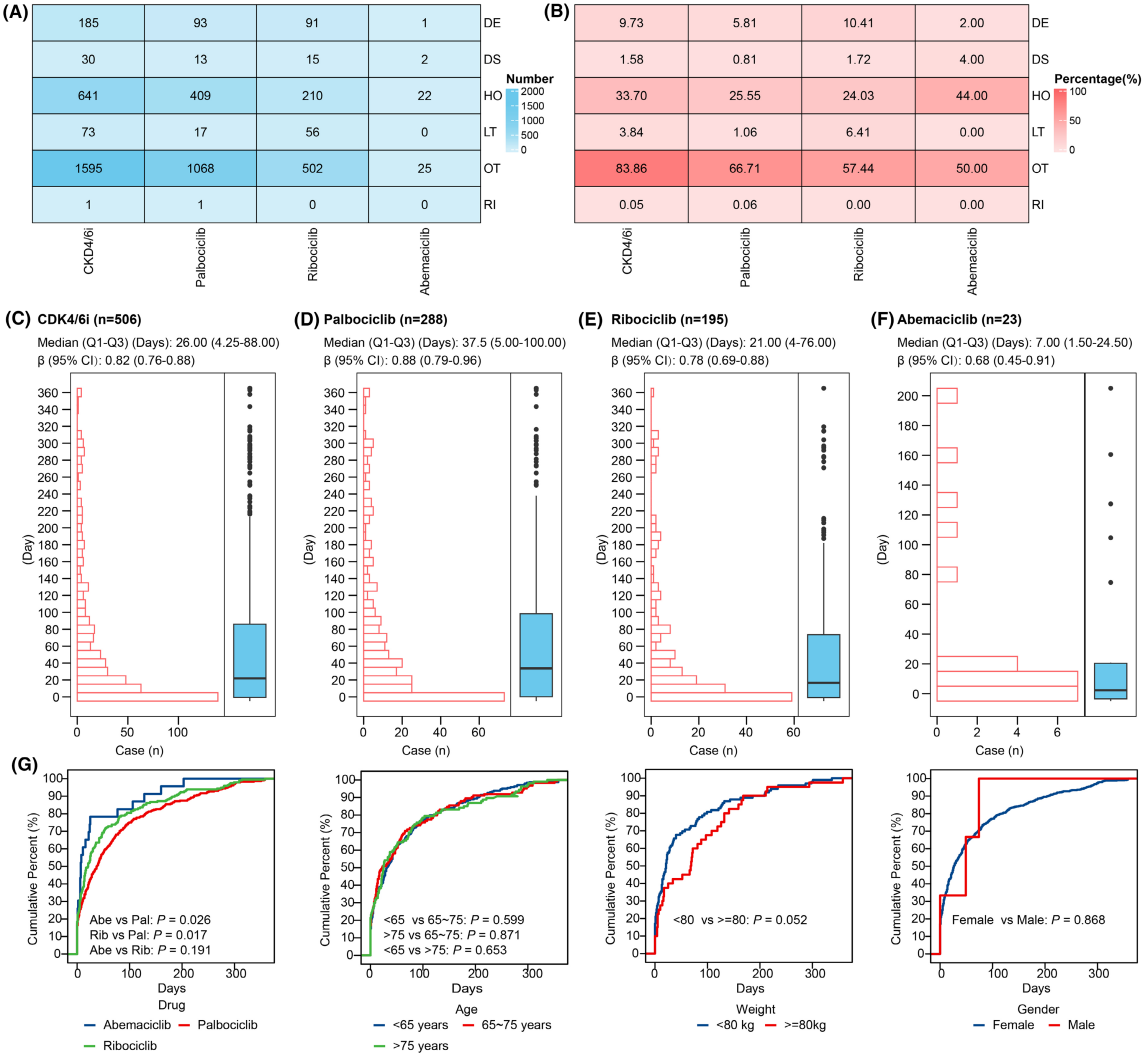
Outcomes of cases with CDK4/6i‐related PAEs and time‐to‐onset analysis. (A) The number of serious outcomes observed in cases of CDK4/6i‐related PAEs. (B) The percentage of serious outcomes observed in cases of CDK4/6i‐related PAEs. (C–F) Histograms and boxplots depict the onset time distribution of CDK4/6i‐related PAEs in case with CDK4/6i (C), palbociclib (D), ribociclib (E) and abemaciclib (F), respectively. (G) The cumulative distribution curves show the onset time of CDK4/6i‐related PAEs in different subgroups. DE, Death; DS, Disability; HO, Hospitalization; LT, life‐threatening; OT, Other Serious; RI, Required intervention to prevent permanent impairment.

We assessed the potential harm of CDK4/6i‐related PAEs by examining the occurrence of serious outcomes in CDK4/6i‐related PAEs cases, including death, hospitalization, life‐threatening situations, disabilities, interventions required to prevent permanent impairment, and other serious outcomes. The number and percentage of serious outcomes were shown in Figure 5A,B, respectively. In 1902 cases with reported outcomes, the mortality, disabilities, and hospitalization rates were 9.73%, 1.58%, and 33.70%, respectively. Among the three CDK4/6i, the ribociclib group had the highest mortality rate of 10.41%, followed by the palbociclib and abemaciclib groups. The abemaciclib group had the highest rate of disabilities, with a rate of 4.00%, followed by the ribociclib and palbociclib groups. The abemaciclib group showed the highest rate of hospitalization at 44.00%, while the palbociclib and ribociclib groups had rates of 25.55% and 24.03%, respectively.

A total of 506 cases were included in the time‐to‐onset analysis. Among these cases, 66.80% experienced CDK4/6i‐related PAEs within the first 2 months of initiating CDK4/6i treatments, with a median onset time of 26.00 days (Q1–Q3: 4.25–88.00) (Figure 5C). Additionally, 17.19% of cases experienced CDK4/6i‐related PAEs on the same day of initiating CDK4/6i treatments. The median onset time for palbociclib was 37.50 days (Q1–Q3: 5.00–100.00), for ribociclib was 21.00 days (Q1–Q3: 4.00–76.00), for abemaciclib was 7.00 days (Q1–Q3: 1.50–24.50) (Figure 5D–F). The upper limit of the 95% CI of β in the WSP tests for CDK4/6i and each drug was less than 1.

We then analyzed the differences in onset time among different subgroups. As shown in Figure 5G, there were significant differences in onset time only between cases with different drugs, while there were no significant differences in onset time among cases with different gender, age, or weight. Specifically, the onset time of CDK4/6i‐related PAEs for palbociclib was significantly higher than that of ribociclib and abemaciclib.

## 4 DISCUSSION

Pre‐marketing clinical trials may overlook rare AEs due to limitations such as limited follow‐up duration, small sample sizes, and strict patient selection criteria. Post‐marketing surveillance in the real world is crucial for detecting and addressing any overlooked safety concerns associated with approved drugs.^28^ The FAERS aids FDA's post‐market surveillance for all approved drugs and biologics. It's open to researchers for pharmacovigilance and pharmacoepidemiologic studies. Based on the FAERS, previous pharmacovigilance studies have elucidated the relationship between CDK4/6i and several AEs, including thromboembolism,^29^ hematopoietic cytopenia,^30^ skin toxicities^31^ and interstitial lung disease.^32^

It is generally accepted that patients with breast cancer are susceptible to psychiatric illnesses, such as depression, anxiety and sexual dysfunction.^10^ With the increasing indications of CDK4/6i in breast cancer, there is an expectation for a continued rise in the usage of CDK4/6i in the future. However, to our knowledge, there is currently a lack of systematic exploration into the association between CDK4/6i and PAEs. Therefore, in this work, we constructed the spectrum of PAEs associated with CDK4/6i and explored the clinical characteristics of cases with CDK4/6i‐related PAEs. Our analysis of the FAERS showed that PAEs reported in 6.72% of the CDK4/6i reports during the period from 2015 to 2023. Palbociclib was approved for marketing in 2015, while ribociclib and abemaciclib were both approved in 2017. Consequently, with the exception of a slight decrease in 2021, the number of CDK4/6i reports in the FAERS database steadily risen from 2015 to 2023. Moreover, the proportion of CDK4/6i reports with PAEs exhibited an upward trend, rising from 3.16% in 2015 to 9.55% in 2023. This underscores the significance of paying close attention to PAEs in patients undergoing CDK4/6i treatment.

We found that out of 182 PAEs reported in CDK4/6i reports, a total of 102 PAEs were reported in no fewer than three cases. Insomnia, anxiety, and depression were the top three PAEs, reported in 17.09%, 11.94%, and 9.14% of cases, respectively. These proportions were close to those documented in clinical trials.^5^, ^6^, ^33^ Taken together, 17 PAEs were categorized as CDK4/6i‐related PAEs, with eating disorder had the highest ROR value among them. Five AEs, including insomnia, stress, eating disorder, depressed mood, and sleep disorder, were very common, each with a prevalence exceeding 10%. Consistent with prior research based on VigiBase,^16^ our research suggested that ribociclib and palbociclib may be depressogenic drugs, while abemaciclib did not show any significant signal for depression symptoms. In addition to this, we found that there were other differences in the profiles of PAEs among different CDK4/6i. In reports of ribociclib, palbociclib, and abemaciclib, the proportions of cases with PAEs were 7.92%, 6.97%, and 2.98%, respectively. The disproportionality analysis indicated that ribociclib and palbociclib had 19 and 18 significant risk signals for PAEs respectively, while abemaciclib only had two. Furthermore, ribociclib treatment showed the strongest association with the occurrence of CDK4/6i‐related PAEs, followed by palbociclib, whereas abemaciclib was not significantly associated with the occurrence of CDK4/6i‐related PAEs. Indeed, previous studies have also reported differences in other toxicity profiles, such as hepatic toxicity, gastrointestinal toxicity, and hematologic toxicity, among the three CDK4/6i.^34^

Our study indicated that in 94.95% of cases, CDK4/6i‐related PAEs did not occur independently, but rather occurred concurrently with other AEs. More specifically, fatigue, nausea, and white blood cell count decreased were observed in 47.66%, 28.00%, and 21.20% of cases, respectively. This was consistent with expectations, as these three AEs were among the most commonly reported in CDK4/6i clinical trials.^35^ It was generally believed that fatigue was a physical disorder, not a psychological illness. However, previous research has shown a strong correlation between chronic fatigue and psychiatric disorders, with up to two‐thirds of individuals experiencing fatigue lasting over 6 months likely to have a concurrent psychiatric disorder.^36^ Those experiencing fatigue without any accompanying psychiatric conditions exhibited heightened levels of specific psychological symptoms and were at an increased risk of developing psychiatric disorders in the future.^37^ Furthermore, gastrointestinal symptoms including nausea, vomiting and constipation were frequent features of eating disorders.^38^ Salvioli et al. has documented that among patients with eating disorders, 96% reported postprandial fullness, 90% reported abdominal distention, and over half complained of abdominal pain, gastric distension, early satiety, and nausea.^39^

We attempted to identify the influencing factors for CDK4/6i‐related PAEs and found that female gender, younger age, and overweight patients are at a higher risk of experiencing CDK4/6i‐related PAEs. It is worth noting that breast cancer primarily occurs in females, so while CKD4/6i can also be indicated for male breast cancer, the limited number of male patients contributes to gender differences in PAEs to some extent. Furthermore, researches have indicated that various psychiatric disorders, including eating disorders,^40^ insomnia,^41^ anxiety^42^ and depression,^43^ were more prevalent among females compared to males. Studies have documented that age affects mental disorders associated with breast cancer, with younger women exhibiting significantly higher rates of mental disorders compared to older women,^44^, ^45^ which aligns with our research. The body mass index (BMI) has been found to be associated with certain toxicities of CDK4/6i, with higher BMI was associated with a significant decrease in neutropenia.^46^ However, we found that patients weighing over 80 kg were 1.17 times more likely to experience CDK4/6i‐related PAEs compared to those weighing less than 80 kg. This was in line with existing research, which suggested that individuals with obesity faced a substantially heightened risk of developing mood or anxiety disorders.^47^

Interestingly, the mortality rates reported for cases with CDK4/6i‐related PAEs in the ribociclib, palbociclib, and abemaciclib groups were 10.41%, 5.81%, and 2.00% respectively. It was widely acknowledged that individuals with mental illnesses experience higher all‐cause and cancer‐specific mortality rates compared to those without mental illnesses among patients with breast cancer.^48^, ^49^ However, mortality rates were also influenced by factors such as demographic characteristics of the medication‐taking population and social environment, as well as the severity of the primary disease and the number of comorbid conditions. Therefore, more rigorous designed trials will be needed to study the correlation between the occurrence of CDK4/6i‐related PAEs and mortality. The median interval between the start of CDK4/6i treatment and the onset of PAEs was 26.00 days. Notably, 17.19% of cases experienced PAEs on the same day after initiating CDK4/6i treatment, while 66.80% of cases occurred within the 2 months of treatment. Study has documented that the median time from the first dose of palbociclib to the appearance of neutropenia was 15 days,^50^ which was earlier than the occurrence of CDK4/6i‐related PAEs in the palbociclib group in our study. The median time to onset for diarrhea in the abemaciclib group was 6 days in the MONARCH‐2 study, similar to the median time for the occurrence of CDK4/6i‐related PAEs in this study, which was 7 days.^51^ Moreover, in all WSP tests, the upper limit of the 95%CI for the *β* was less than one, indicating that the risk of CDK4/6i‐related PAEs gradually decreased over time.

The specific mechanism by which CDK4/6i induce psychiatric disorders is not yet clear. However, it is generally accepted that adult hippocampal neurogenesis contributes to emotional regulation, and its dysregulation is linked with psychiatric disorders.^52^ From a pharmacological perspective, CDK4/6i disrupt the transition of the cell cycle from G1 to S phase by suppressing the kinase activity of the CDK4/6‐Cyclin D complex, thereby impeding the phosphorylation of Rb protein, a critical step in cell cycle progression.^53^ Thus, the non‐cell‐specific nature of CDK4/6i implies their potential interference not only with the cell cycle of cancer cells but also with other cells, such as neural precursor cells in hippocampus, which are pivotal for neurogenesis. Research has documented that CDK4/6 activity, particularly their phosphorylation of Rb, is crucial for cell cycle regulation in primary neural precursor cells, and mutations in CDK4/6 can lead to mitotic arrest in these cells.^54^ The cyclin D2, which forms an active complex with CDK6 in the G1 phase of the cell cycle, plays a pivotal role in adult neurogenesis.^55^ Therefore, we speculate that CDK4/6i inhibit the CDK4/6‐Cyclin complex D in neural precursor cells, potentially leading to impaired neurogenesis and consequently inducing psychiatric disorders. Additionally, several previous studies may explain why abemaciclib exerts less neuropsychiatric toxicity compared to the other two CDK4/6i. A notable study using knockout mice for Cdk4 and Cdk6 to explore their effects on adult neurogenesis revealed a predominant role for Cdk6, whereas Cdk4 showed less pronounced involvement in regulating neurogenesis.^56^ Abemaciclib, when compared to palbociclib and ribociclib, demonstrates significant selectivity for CDK4, being about five times more potent against CDK4 than CDK6.^57^

The present study, like other pharmacovigilance studies based on spontaneous reporting systems, acknowledged certain limitations. Firstly, duplication was a prevalent issue in the FAERS database. To tackle this concern, our analysis not only involved excluding duplicate reports based on CASEID but also filtered out cases with identical characteristics including gender, age, reporting country, event date, AEs, and prescribed drugs. However, given that over 70% of reports lacked at least one of these characteristics, identifying all duplicated cases became challenging. Secondly, the fact of under‐reporting and misreporting existed in the FAERS database. For instance, the under‐reporting of cases from countries beyond the USA restricted the generalizability of the conclusions drawn in this study, and inaccuracies or omissions in recording START_DT and EVENT_DT potentially introduced biases into the time‐to‐onset results. Thirdly, it was indeed challenging to discern whether psychiatric disorders in each case were induced by breast cancer, other drugs, or by CDK4/6i. Therefore, to enhance the reliability of this study, we exclusively included reports where CDK4/6i were listed as the PS drug. This decision was made under the assumption that reporters had already assessed the relationship between drug use and AEs in each case and deemed CDK4/6i as the most likely medication to elicit AEs in the respective reports. Finally, patient race was an important factor influencing AEs. Since patient race information was not provided in individual case safety reports in the FAERS, we were unable to analyze the impact of race on CDK4/6i‐related PAEs in our study. However, given the scarcity of research on CDK4/6i‐related PAEs, our analysis of a large database offered comprehensive insights into the relationship between CDK4/6i and PAEs. Our findings provided valuable evidence to support further research and clinical practice in this area, ultimately enhancing patient care and safety in the context of CDK4/6i therapy. Nevertheless, as this study is retrospective in nature, in our future research, we will design a prospective study to compare the incidence and severity of PAEs among the three CDK4/6i.

## 5 CONCLUSIONS

In conclusion, leveraging real‐world data from the FAERS database, this pharmacovigilance study conducted a comprehensive investigation and delineated the spectrum of PAEs to CDK4/6i. Our analysis identified 17 categories of PAEs highly associated with CDK4/6i by conducting disproportionality analysis. There were differences in the profiles of CDK4/6i‐related PAEs among different drugs, with ribociclib presented the highest risk signal, while abemaciclib showed no significant signal. Hence, healthcare providers should consider these distinctions when selecting medications and inform patients about the potential risk of PAEs, particularly those with pre‐existing psychiatric disorders. However, large‐scale, population‐based prospective studies are crucial for accurately determining the actual incidence of CDK4/6i‐related PAEs and fully elucidating the potential biological mechanisms and risk factors to enhance risk management strategies.

## AUTHOR CONTRIBUTIONS

FX and ZY conceived and designed the study. ZX and JC performed the data analysis and wrote the manuscript. SW, TZ, CL and JD collected the data and revised the manuscript. All authors contributed to the article and approved the final submitted manuscript.

## FUNDING INFORMATION

This work was supported by the Shanghai Fengxian District Science and Technology Committee (No.20221206).

## CONFLICT OF INTEREST STATEMENT

The authors declare that there is no conflict of interest associated with this study.

## Supporting information


Figures S1–S2.

Tables S1–S2.

## Data Availability

The raw data included in this work were downloaded from the FAERS database at https://fis.fda.gov/extensions/FPD‐QDE‐FAERS/FPD‐QDE‐FAERS.html.
