# Supplementary material for: Spectrum of psychiatric adverse reactions to cyclin‐dependent kinases 4/6 inhibitors: A pharmacovigilance analysis of the FDA adverse event reporting system
Source: CNS Neurosci Ther. 2024 Jul 15;30(7):e14862. doi: 10.1111/cns.14862 (PMC11250168; doi:10.1111/cns.14862)
Supplement: Supplementary file 1 — Figures S1–S2. [file CNS-30-e14862-s002.docx]

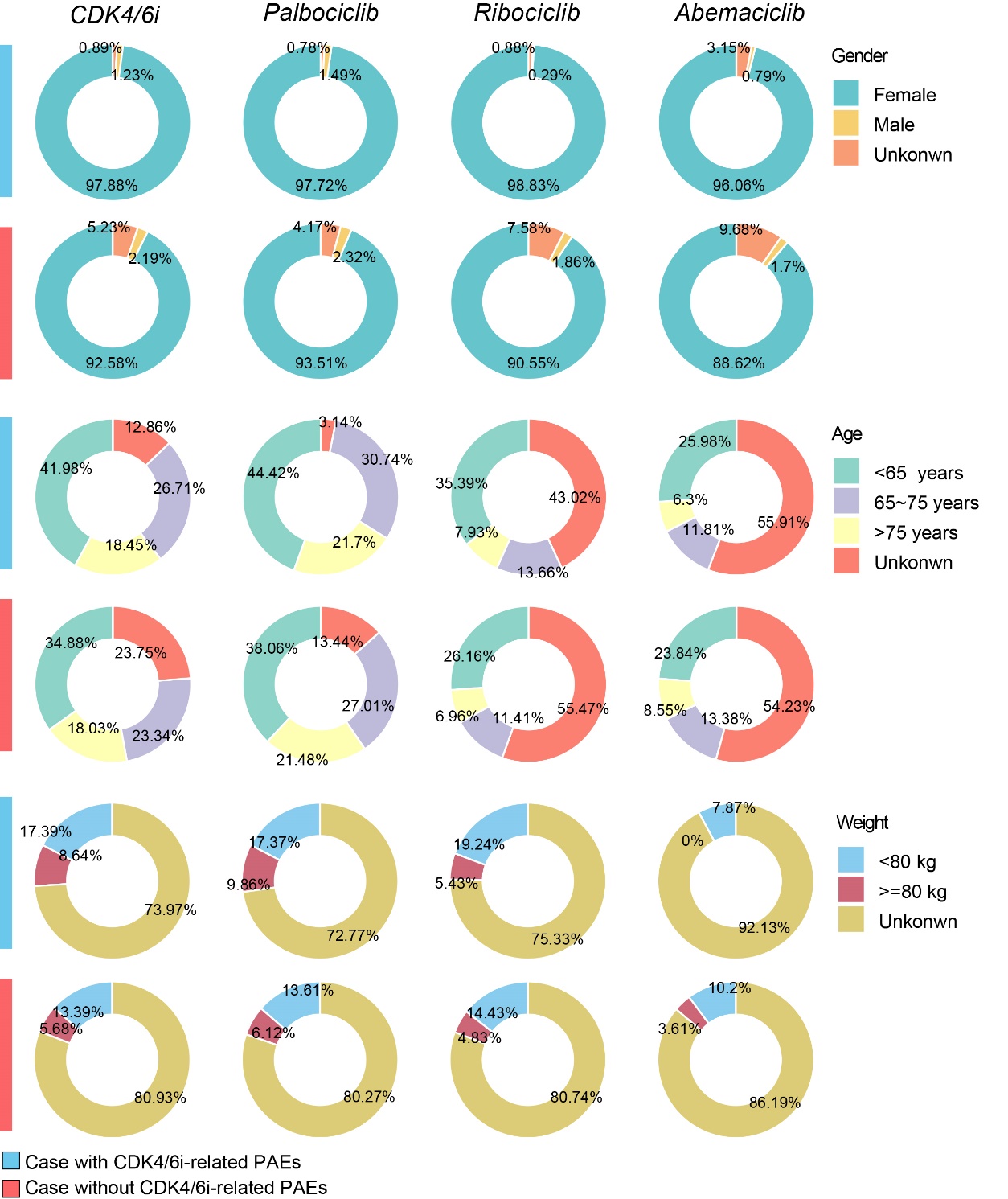


**FIGURE S1** The distribution of age, gender, and weight among cases with and without CDK4/6i-related PAEs


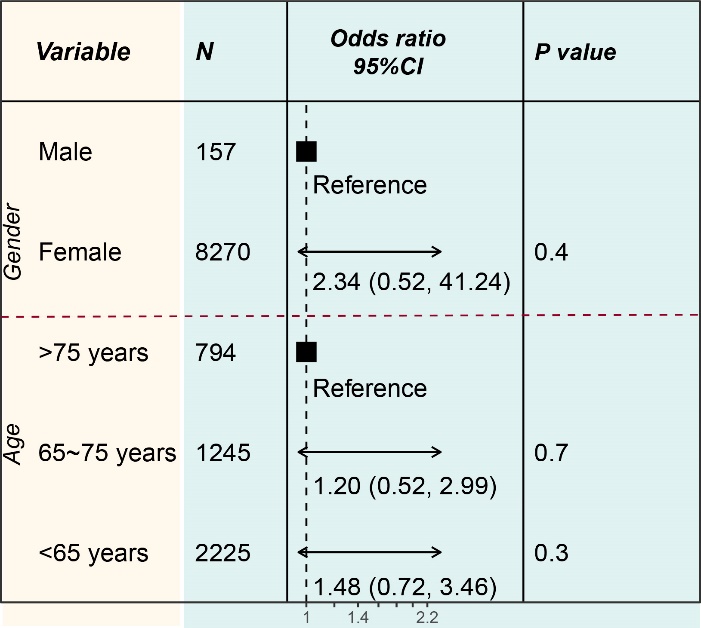


**FIGURE S2** Forest plot shows the outcomes of univariate logistic regression analysis concerning demographic factors influencing CDK4/6i-related PAEs for abemaciclib cases
